# Moderately HRT vs. CRT for localized prostate cancer using image-guided VMAT with SIB: evaluation of acute and late toxicities

**DOI:** 10.1007/s00066-020-01589-w

**Published:** 2020-02-10

**Authors:** Stratos Vassis, Beatrice Nöldeke, Hans Christiansen, Christoph A. von Klot, Roland Merten

**Affiliations:** 1 Department of Radiation Oncology, Hannover Medical School, Carl-Neuberg-Str. 1, 30625 Hanover, Germany; 2 Department of Urology, Hannover Medical School, Carl-Neuberg-Str. 1, 30625 Hanover, Germany; 3 Institute for Environmental Economics and World Trade, Leibniz University, Königsworther Platz 1, 30167 Hanover, Germany

**Keywords:** Localized prostate cancer, Moderately hypofractionated radiotherapy, Simultaneous integrated boost, Genitourinary toxicity, Gastrointestinal toxicity, Lokal begrenztes Prostatakarzinom, Moderat hypofraktionierte Strahlentherapie, Simultan integrierter Boost, Urogenitale Toxizität, Gastrointestinale Toxizität

## Abstract

**Purpose:**

This retrospective study aims at investigating the effects of moderately hypofractionated radiation therapy (HRT) on acute and late toxicities as well as on early biochemical control and therapeutic efficiency compared to conventional radiation therapy (CRT) in prostate cancer.

**Patients and methods:**

We analyzed 55 HRT patients irradiated with the total dose of 60 Gy in 20 fractions delivered over 4 weeks. These patients were compared to a control group of 55 patients who received CRT with a total of <78 Gy in 37–39 fractions delivered over circa 8 weeks. External beam radiation therapy (EBRT) was conducted using daily image-guided (cone beam CT) volumetric modulated arc therapy (VMAT) and a simultaneously integrated boost (SIB) for both groups to protect the rectum. Acute toxicities were evaluated according to Common Terminology Criteria for Adverse Events (CTCAE) v5, whereas chronic toxicities were assessed in accordance with LENT-SOMA. Patient traits were compared by implementing t‑tests and Wilcoxon–Whitney tests for continuous variables, whereas discrete characteristics were evaluated by applying two-tailed Fisher’s exact tests. In addition, we calculated average treatment effects (ATE). Thereby, propensity score matching (PSM) based on nearest-neighbor matching considering age, comorbidities, and risk stratification as covariates was applied. The statistical analysis was conducted using Stata 14.2 (StataCorp LLC, TX, USA).

**Results:**

As confirmed by the descriptive tests, the ATE revealed that the intensity and occurrence of urinary frequency (*p* = 0.034) and proctitis (*p* = 0.027) significantly decreased for the HRT group, whereas all other acute toxicities did not differ significantly between the HRT and CRT groups. For late toxicities, neither statistical tests nor ATE estimation showed significant differences. Also, no significant difference was found regarding the decrease in prostate specific antigen (PSA) after a median follow-up of 13 months (range 2–28 months), which indicates biochemical freedom from progression.

**Conclusion:**

HRT offers several medical and economic advantages and should therefore be considered as a useful alternative to CRT.

## Introduction

Whilst a variety of different treatment options for localized prostate cancer (LPCa) exist, external beam radiation therapy (EBRT) is considered one of the primary therapies for patients of all risk classifications [1, 2]. EBRT aims at controlling tumor growth while keeping acute and late adverse events to a minimum and ensuring biochemical progression-free outcomes [3]. In recent years, a variety of technical improvements of EBRT such as volumetric intensity-modulated radiotherapy (VMAT/IMRT) and image-guided radiotherapy (IGRT) have been developed. These advancements enable an escalation of the dose applied to the prostate. In this context, Viani et al. found a significant impact of dose escalation on biochemical control (BC) in all risk groups [4]. However, the toxicities of surrounding tissues restrict the extent of dose escalation [5]. In order to protect adjacent sensitive tissues such as the bladder and rectum, IMRT can additionally be combined with simultaneous integrated boost (SIB), allowing distinct radiation doses to be delivered to the cancer site and bordering organs during a single session [6–8].

Fractionation schedules of radiotherapy can either be conventionally fractionated or hypofractionated. Hypofractionated radiotherapy (HRT) generally applies single doses of 2.4–3.1 Gy directed at the prostate and seminal vesicle, whereas effective single doses are lower for conventional radiation therapy (CRT) [5, 9]. The raise in the daily single dose for HRT is based on a low α/β ratio estimate for prostate cancer (PCa), which is assumed to cause a significantly higher sensitivity towards increased fraction dose. Whereas Brenner et al. [10] estimate the α/β ratio for PCa to range from 1 to 1.8 Gy, Vogelius and Bentzen [11] estimate the α/β ratio to be 1.2 Gy (95% CI: 0.8–1.7 Gy) and 2.7 Gy (95% CI: 1.6–3.8 Gy), concluding that moderate HRT is consistent with a low value of the α/β ratio. In contrast, the value for adjacent organs such as bladder or rectum is 3–5 Gy [12].

Recent studies comparing hypofractionation and conventional fractionation in phase III trials conclude that HRT leads to similar or even improved late genitourinary (GU) and gastrointestinal (GI) toxicities [13, 14]. In addition, HRT confers improved availability, decreased costs, and shortened treatment duration, and thereby provides relief, especially for patients of advanced age or those suffering from multiple comorbidities. Countries such as the United States of America, Canada, United Kingdom, the Netherlands, and Italy have already established HRT as the clinical standard for radiotherapy of LPCa [15–18].

We conducted a non-randomized retrospective clinical trial to verify that moderate HRT is a treatment alternative with high potential to improve cancer control by evaluating its effects on acute and late toxicity in a cohort of 110 patients. To our knowledge, this study is the first HRT trial for prostate cancer conducted in Germany. The achieved decrease in toxicities reflects its high practical relevance.^1^

## Patients and methods

### Patients

The present retrospective analysis examined 55 patients diagnosed with LPCa and treated with moderately hypofractionated radiotherapy in the time period between July 2016 and December 2018 at the Institute of Radiation Therapy and Special Oncology, Hanover Medical School.

Before consenting to enroll in the study the patients were informed about the current scientific status of HRT and possible side effects with respect to genitourinary toxicities. The medical briefing took place in accordance with the interdisciplinary German S3 guideline for PCa. Prior to proposing HRT to patients, risk factors such as comorbidity, risk stratification, age, and physical performance were taken into consideration. To assess the impact of HRT, we compared the HRT patients to a control group irradiated with CRT. Patients with radical prostatectomy, lymphadenectomy, prior history of radiotherapy, evidence of pelvic nodal disease, and presence of distant metastases were excluded from the study; patients with biopsy-proven PCa T1b to T3b defined by the TNM system without evidence of distant metastasis were found eligible. The previously larger sample of CRT patients was further filtered using characteristics such as D’Amico risk classification for PCa, Charlson comorbidity index (CCI), androgen deprivation therapy (ADT), and age, to ensure comparability and resulting high quality of matching (CRT *n* = 55).

The share of patients classified as high (intermediate) risk according to D’Amico classification was 46% (49%) in the HRT group and 42% (51%) for CRT patients. The most frequently occurring diseases captured by the CCI [19] included diabetes, myocardial infarct, congestive heart failure, peripheral vascular disease, moderate and severe renal diseases, and additional tumors with similar distributions in both groups (Table 1).

**Table 1:** Patient characteristics by treatment regimen

Characteristics	Conventional fractionation (72–78 Gy in 2.0 Gy fractions)	Hypofractionation (60 Gy in 3.0 Gy fractions)
*No. of patients*	55	55
*Age, years*		
<65	2 (3.6)	3 (5.4)
65–74	12 (21.8)	8 (14.5)
75–79	30 (54.5)	20 (36.4)
≥80	11 (20.0)	24 (43.6)
*Tumor stage*		
T1–T1c	44 (80.0)	45 (81.8)
T2 a–b	5 (9.1)	6 (10.9)
≥T2c	6 (10.9)	4 (7.3)
*Gleason score*		
≤6	9 (16.4)	7 (12.7)
7	27 (49.1)	23 (41.8)
≥8	19 (34.5)	25 (45.5)
*D’Amico risk classification*		
Low	4 (7.3)	3 (5.5)
Intermediate	28 (50.9)	27 (49.1)
High	23 (41.8)	25 (45.5)
*Charlson comorbidity index (CCI)*		
≤3	26 (47.3)	18 (32.7)
4	15 (27.3)	14 (25.5)
5	7 (12.7)	8 (14.6)
6	3 (5.5)	8 (14.6)
≥7	4 (7.3)	7 (12.7)
*ADT*		
Yes	25 (45.5)	31 (56.4)
No	30 (54.5)	24 (43.6)
*Nicotine*		
Yes	3 (5.5)	10 (18.2)
*Alcohol*		
Yes	15 (27.3)	17 (30.9)
*Medication*		
Anticoagulants	21 (38.1)	16 (29.1)
Antiplatelet agents	13 (23.6)	20 (36.4)
Non-antithrombotic drugs	41 (74.5)	40 (72.7)

Data are given in no.; % in parenthesis. Table shows the baseline characteristics in HRT and CRT groups

*ADT* androgen deprivation therapy, *HRT* hypofractionated radiation therapy, *CRT* conventional radiation therapy

### Methods

EBRT for LPCa employed intensity-modulated VMAT combined with SIB. The specific doses were calculated based on Monte Carlo treatment planning simulations and are listed in Table 2. The HRT group received 50.0 Gy directed to the prostate and seminal vesicle with a single dose of 2.5 Gy and an additional SIB applied only to the prostate with the effective single dose of 3.0 Gy summing up to a total of 60.0 Gy delivered in 20 fractions over 4 weeks (EQD2 77.1 Gy, α/β = 1.5 Gy). In contrast, CRT patients obtained a total dose of 50.4–59.4 Gy delivered in 1.8–2 Gy fractions to the prostate and seminal vesicle combined with a SIB to the prostate to 66 Gy, followed by an additional boost (sequential boost) to the prostate up to 72–78 Gy delivered in 2.0 Gy fractions over 8 weeks total treatment time.

**Table 2:** Treatment characteristics by regimen

Variable	CRT	HRT
*No. of patients*	55	55
*Dose prescriptions, Gy*		
60	0	55 (100%)
72	2 (3.6%)	0
74	29 (52.7%)	0
76	23 (41.8%)	0
78	1 (1.8%)	0
*Single dose, Gy 5/week*	2.0	3.0
*Boost modality*		
SIB but without additional boost	0	55 (100%)
SIB + additional boost (SQ)	55 (100%)	0
*Average of IGRT, n/week*	2.3	5.0
*PTV (prostate* *+proximal vesicle, ccm)*	229.9	204.4
*PTV (SIB prostate, ccm)*	184.8	148.0
*Dmean, rectum, Gy*	37.4	27.7
*Dmean, bladder, Gy*	34.6	24.8
*Overall treatment time, days*	56	29

*CRT* conventional radiation therapy, *HRT* hypofractionated radiation therapy, *SIB* simultaneous integrated boost, *SQ* sequentially boost, *IGRT* image-guided radiotherapy, *PTV* planning target volume

All HRT patients received daily IGRT, whereas the vast majority of the CRT group obtained IGRT 2.3 times per week on average. IGRT was provided including a cone beam CT (CBCT) mounted on the gantry of a linear accelerator (VersaHD) using an Elekta x‑ray volume imager (XVI).

**Fig. 1:**
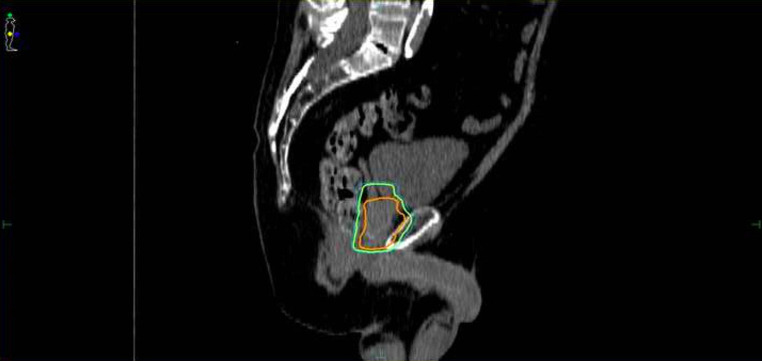
Reduction of planning target volume (PTV) of “prostate and proximal vesicle” (*green*) to PTV of “simultaneous integrated boost (SIB) prostate without proximal vesicle” (*orange*). Delineation of rectum as a region of interest is not included in the graphic to improve visualization of the relevant anatomic features

In our study, gross tumor volume (GTV) equals the prostate since the tumor burden from MRI and PSMA-PET was not available for all patients. Safety margins comprised 3 mm from GTV to clinical target volume (CTV) and 5 mm from CTV to planning target volume (PTV). The larger PTV includes prostate and seminal vesicle, whereas the SIB-PTV only contained the prostate without the seminal vesicle to protect the rectum as shown in Fig. 1 [20]. The mean SIB-PTV for the prostate gland without margins was 148.0 cm^3^ for the HRT group and 184.8 cm^3^ for the CRT group. Furthermore, the delivered mean dose to the urinary bladder (rectum) for the HRT arm was 24.8 Gy (27.7 Gy) and 34.6 Gy (37.4 Gy) for the CRT arm. The overall treatment time differed strongly between the groups: for the CRT group it amounted to up to over 55 days on average, whereas it lasted only 29 days for the HRT group.

Whilst undergoing radiation therapy, patients’ adverse events were controlled weekly. After treatment completion, patients were examined firstly after approximately 3 months, then in a yearly cycle. Acute adverse events were categorized retrospectively according to CTCAE version 5 [21], whereas the chronic adverse events were assessed in accordance with LENT-SOMA tables [22]. Acute and late GI/GU toxicities were defined as primary endpoints. The secondary endpoints were given by BC.

### Statistical evaluation

Descriptive statistics were generated to characterize the study cohort. For continuous variables, the groups were compared by implementing the Student t‑test and Wilcoxon–Mann–Whitney-test; for categorical and binary variables the two-sided Fisher’s exact test was applied, because the expected frequencies are rather small and the Fisher’s exact test does not require a certain sample size [23]. In addition, the average treatment effect (ATE) was calculated to compare toxicities between the treatment and control groups. Since the ATE can be biased in nonrandomized observational trials like the present study, propensity score matching (PSM) is applied to mimic a randomized trial and enable unbiased analysis [24, 25]. Thereby, the propensity score (PS) describes the probability of treatment assignment dependent on observed patients’ traits as covariates, namely D’Amico risk stratification, ADT, CCI, and age. A logit model estimates the PS [24, 25]. In the next step, patients from the treatment and control groups are paired according to nearest-neighbor matching: treated patients are matched to those from the control group who are closest in terms of their PS with a caliper corresponding to 26% of the standard deviation of the PS. Lastly, a comparison of the matched patients provides an unbiased estimate of the ATE between the two groups [26]. To ensure robustness, we checked the pseudo‑R^2^, changed the nearest neighbor ratio, restricted replacement, and performed local linear regressions and kernel regressions. As an additional robustness check, we estimated the ATE based on inverse probability weights. Furthermore, we evaluated the area of common support, maximum and minimum values, and the balancing property of the PS. The statistical analysis was conducted using Stata 14.2 (StataCorp LLC, TX, USA).

## Results

### Patients

The HRT group received 50.0 Gy (single dose 2.5 Gy) to the prostate and seminal vesicle, integrating an SIB to the prostate at 60 Gy (single dose 3.0 Gy). The CRT group completed treatment as planned, with one patient pausing treatment due to an acute abdominal emergency. No data regarding the late toxicities were collected for two patients of each group during follow-up.

### Response and local control

To capture the secondary endpoints, PSA values were measured after 3 months, 6 months, 12 months, and later than 1 year. Patients receiving ADT were excluded from evaluation of secondary endpoints to avoid bias in the results. Amongst those patients not obtaining ADT, PSA bounces occurred in all cases. After 3 months, values for PSA bounces were available for all patients and dropped by 6.1 ng/ml for the CRT group and 5.7 ng/ml for the HRT group. Values measured after at least 12 months showed a decrease of 7.6 for the CRT group and 7.1 for the HRT arm. The t‑test and Wilcoxon–Mann–Whitney test revealed no significant differences between the treatment and control groups with respect to freedom from biochemical or clinical failure according to the Phoenix definition [27]. Mean follow-up was 16 months for the CRT group and 10 months for the HRT group.

### Toxicity

We tested differences in acute GI toxicities (proctitis, colitis, diarrhea, defecation frequency, colonic obstruction) and acute GU toxicities (urinary frequency, dysuria, noninfective cystitis) using CTCAE (Table 3 and 4). Furthermore, we collected data regarding radiation-related acute dermatitis and fatigue. For late adverse GU and GI toxicities we stratified subcategories according to LENT-SOMA tables as follows: bladder/urethra, skin and subcutaneous tissue, small intestine, and colon.

**Table 3:** Acute GI/GU toxicity by grade and treatment

	HRT			CRT		
Acute toxicity	Grade 1	Grade 2	Grade 3	Grade 1	Grade 2	Grade 3
Proctitis	5 (9.1)	0	0	6 (10.9)	7 (12.7)	0
Diarrhea	2 (3.6)	1 (1.8)	0	3 (5.5)	0	0
Colitis	2 (3.6)	1 (1.8)	0	3 (5.5)	0	0
Colonic obstruction	1 (1.8)	0	0	1 (1.8)	2 (3.6)	0
Defecation frequency	1 (1.8)	0	0	0	0	0
Urinary frequency	20 (36.4)	2 (3.6)	0	25 (45.5)	7 (12.7)	1 (1.8)
Cystitis non-infective	3 (5.5)	2 (3.6)	1 (1.8)	1 (1.8)	2 (3.6)	1 (1.8)
Dysuria	8 (14.5)	0	0	14 (25.5)	0	0
Fatigue	3 (5.5)	0	0	3 (5.5)	0	0
Dermatitis radiation	3 (5.5)	0	0	5 (9.1)	0	0

Data are given as no.; % in parenthesis

**Table 4:** Late GI/GU toxicity by grade and treatment

	HRT				CRT			
Late toxicity	Grade 1	Grade 2	Grade 3	Grade 4	Grade 1	Grade 2	Grade 3	Grade 4
Skin	1 (1.8)	0	0	0	1 (1.8)	0	0	0
Small intestine	1 (1.8)	0	0	0	3 (5.5)	1 (1.8)	0	1 (1.8)
Colon	8 (14.5)	0	0	0	5 (9.1)	1 (1.8)	0	1 (1.8)
Bladder/urethra	9 (16.4)	2 (3.6)	2 (3.6)	0	12 (21.8)	3 (5.5)	2 (3.6)	0

Data are given as no.; % in parenthesis

Out of the CRT group, 78% patients reported incidences of acute toxicities grades 1–3 compared to 62% of the HRT group. CRT patients suffered from proctitis grade 2 (13%), urinary frequency grade 1 (46%), and urinary frequency grade 2 (13%) more frequently than HRT patients, who reported values of 0%, 36%, and 4%, respectively (Table 3). Acute grade 3 GU toxicities were recorded rarely; only 1 patient from the CRT group experienced urinary frequency, and 1 patient from each group suffered from cystitis. Late toxicities of grade 4 were only found in the CRT arm, as shown in Table 3.

The results illustrated in Table 5 reveal that according to the two-sided Fisher’s exact test, significant differences between the control and treatment groups exist with respect to proctitis (*p* = 0.019) and urinary frequency (*p* = 0.071). However, for none of the other adverse events were statistically significant differences found. For late adverse events, the test did not reveal significant differences between the two groups. Hence, for late adverse events, HRT yields equal results to CRT.

**Table 5:** Average treatment effect and results of two-tailed Fisher’s test

Symptoms	ATE (*p*-value)	Two-tailed Fisher test *p*-value
Proctitis	−0.26** (0.027)**	0.019**
Diarrhea	0.02 (0.696)	1.000
Colitis	−0.01 (0.875)	1.000
Dermatitis radiation	−0.02 (0.720)	0.716
Cystitis noninfective	0.10 (0.293)	0.926
Urinary frequency	−0.30** (0.034)**	0.071*
Dysuria	−0.13 (0.176)	0.233
Defecation frequency	0.02 (0.245)	1.000
Fatigue	−0.02 (0.728)	1.000
Colonic obstruction	−0.04 (0.460)	0.745
Skin	0.01 (0.737)	1.000
Small intestine	−0.08 (0.268)	0.237
Colon	0.01 (0.892)	0.555
Bladder/Urethra	0.01 (0.951)	0.870

Second column: Average treatment effects of HRT compared to CRT, *p*-values in parentheses

Third column: Significance level indicated by *p*-values for differences between HRT and CRT group

*p < 0.1, **p < 0.05

**Fig. 2:**
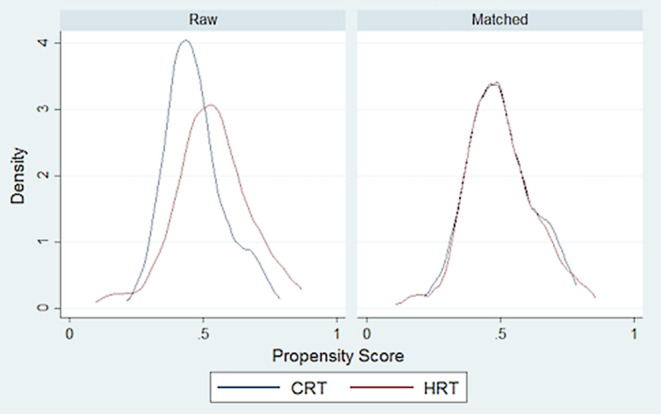
Distribution of propensity score before and after nearest-neighbor matching

**Fig. 3:**
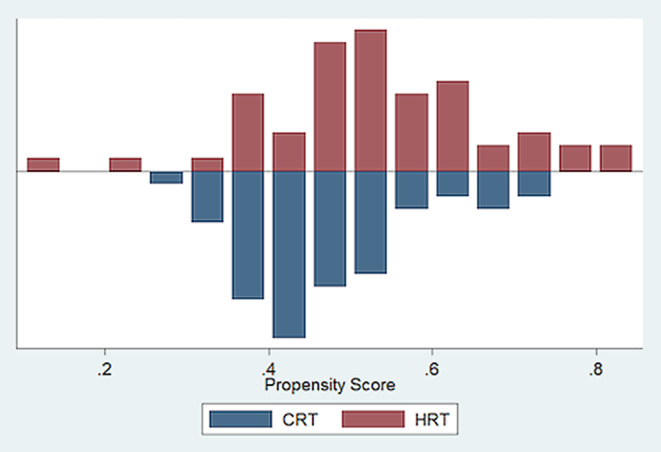
Area of common support and overlap between CRT and HRT groups show balance between the two groups and high quality of matching

Table 5 presents the average treatment effects as calculated based on propensity score matching. A total of six blocks were identified to ensure that the mean propensity score does not differ between treatment and control groups. Fig. 2 illustrates the decline in bias through PSM. Fig. 3 shows that the baseline-recorded characteristics overlap; hence, balance between the two groups exists and an area of common support is given. The results of the ATE support the previous results of the Fisher’s exact tests: as illustrated, differences between the therapy and the control groups are only significant for proctitis and urinary frequency. Specifically, the ATE reveal that occurrence and intensity of proctitis and urinary frequency are lower for the HRT group (*p* = 0.027 and *p* = 0.034, respectively). For all other symptoms (acute as well as late), the differences were not statistically significant and, hence, HRT does not intensify the side effects.

Furthermore, since the larger PTV volume (prostate and seminal vesicle) and SIB-PTV (prostate) differed between HRT and CRT cohort as indicated in Table 2, we tested correlations between occurrence of any toxicity and PTV volumes. Despite a possible clinical causal relation, the analysis revealed no significant correlations in both patient groups.

## Discussion

In our study we compared the treatment effectiveness of moderate HRT performed with modern techniques (IMRT-IGRT-SIB) to CRT by analyzing the occurrence and intensity of acute and late toxicities. The findings showed improved treatment effects regarding irradiation-related acute GI and GU toxicity, namely for proctitis and urinary frequency, whereas HRT proved to be an equivalently efficient treatment method when compared with CRT regarding its effect on all other toxicities. A decrease in PSA values in the follow-up care was achieved for both the HRT and CRT arms, which implies biochemical tumor control in both groups.

Several previous studies have explored the effects of HRT. In the context of a meta-analysis, Datta et al. summarized data from randomized trials with follow-up periods ranging from 6 to 10 years to compare moderate HRT with CRT using doses from 57.0 to 62.0 Gy for the HRT arm and 74.0 to 80.0 Gy for the CRT arm. Datta et al. conclude that in the investigated trials, such as the PROFIT [14], IRE [28–31], RTOG [30], CHHiP [17], and HYPRO [32] studies, HRT leads to similar or even improved results regarding biochemical outcomes and toxicity. Also, Schörghofer et al. [33] report that moderate HRT lowers GU and GI toxicity through all risk classifications. Additionally, the recently published study from Hoffman et al., which includes a follow-up of 8 years, finds improved tumor control through HRT. However, some investigations find the incidence of acute GI toxicity to be significantly higher (between 0.3 and 13.9%) in the HRT group compared to CRT treatment [13, 34].

These outcomes with respect to increased acute GI toxicity refined our study design, which aimed at preventing increased toxicity by implementing an additional SIB to the prostate and modern techniques. To achieve this goal, we supported the radiotherapy entirely with IMRT-IGRT-SIB plans for both groups. Using fractionation schedules of previous studies as an orientation, we applied a modified dose of 50.0 Gy to the prostate and seminal vesicle with an additional 10.0 Gy as a SIB directly to the prostate in 20 fractions (EQD2 77.0 Gy, α/β = 1.8 Gy) over a period of 4 weeks, to reduce rectum and bladder complications.

The control group received a total dose of 50.4–59.4 Gy delivered in 1.8–2 Gy fractions to the prostate and seminal vesicle combined with a SIB to the prostate to 66 Gy, followed by an additional boost (sequential boost) to the prostate up to 72–78 Gy delivered in 2.0 Gy fractions over 8‑weeks’ total treatment time. The implementation of analogous IMRT-IGRT-SIB plans for both groups allowed an efficient treatment cohort comparison.

Regarding dose constraints, Yu [35] and Hoffman et al. predict that smaller dose constraints, especially with respect to the rectal doses, lead to enhanced cancer control and less acute GI toxicity rates. Consequently, we carefully considered the dose constraints for the adjacent organs. We chose dose constraints following the study of Catton et al., but decreased them by around 10% (Table 6 und 7).

**Table 6:** Dose–volume criteria for the HRT group

Volume of interest	Metric	Constraint for planning (Gy)	Mean dose (Gy) reached in treatment group
*Target volume*			
Clinical	D99	≥60	≥60
Planning	D99	≥57	≥57
*Rectal wall*	D50	≤37	27.7
*Bladder wall*	D50	≤37	24.8

**Table 7:** Dose–volume criteria for the CRT group

Volume of interest	Metric	Constraint for planning (Gy)	Mean dose (Gy) reached in treatment group
*Target volume*			
Clinical	D99	≥78	≥78
Planning	D99	≥74	≥74
*Rectal wall*	D50	≤60.8	37.4
*Bladder wall*	D50	≤60.8	34.6

The descriptive analysis based on the two-sided Fisher’s exact test reveals that the occurrence and intensity of urinary frequency as well as proctitis decreased in the HRT group (*p* = 0.071 and *p* = 0.019, respectively). In contrast, the occurrence and intensity of all other late and acute toxicities do not differ significantly between the HRT and CRT arms. The average treatment effects confirmed these results (Table 5) and simultaneously proved the robustness of the findings by counteracting the selection bias persistent in nonrandomized trials through propensity score matching. These results correspond with the findings of other studies [36], and the implementation of SIB and modern techniques and restricted dose constraints further prevents increased GI adverse events. The study adds to the existing state of the art by integrating these modifications, which results in significantly reduced occurrence and intensity of proctitis and urinary frequency.

Several limitations of this study should be considered. The findings of the present analysis predominantly apply to intermediate- and high-risk LPCa patients with multiple comorbidities and high age. Due to advanced patient age and the current data base, the follow-up was relatively short (16 months for the CRT group and 10 months for the HRT group). Furthermore, the number of patients undergoing HRT was comparatively small, which reflects the slow shift towards HRT in Germany. Yet, as proven by statistical analysis, the findings remain robust and offer interesting insights into the efficiency of HRT. Further research could include additional consideration of bladder and rectum volumetric variations throughout treatment, to decrease toxicities as described by Gawish et al. [37] and Grün et al. [38].

## Conclusion

The present study concludes that hypofractionated radiation schedules and modified treatment plans integrating modern techniques and implementing an SIB directly to the prostate lead to reduced toxicity rates and improved tumor control. Furthermore, the smaller number of fractions and higher single dose, which are accompanied by shorter treatment duration, improve patient comfort and compliance. Additionally, decreased health costs enhance accessibility of the treatment. Thus, HRT offers several medical and economic advantages and should therefore be considered as clinical standard, also in Germany.

## Footnotes

1 Parts of the results have been orally presented at the DEGRO Annual Meeting 2019 in Münster.
